# Coagulation biomarkers as prognostic indicators in heart failure: A systematic review and meta-analysis of 14,773 patients

**DOI:** 10.21542/gcsp.2025.41

**Published:** 2025-08-30

**Authors:** Ceria Halim, Billy Putra Teruna, Indah Ramadhani Harahap, Eric Teo Fernando, L. Brianto Christian Nugroho

**Affiliations:** Department of Center of Evidence Based Medicine, Faculty of Medicine, Universitas Sumatera Utara, Medan, Indonesia

## Abstract

Introduction and objectives: Heart failure (HF) patients are at risk of developing thrombosis due to low cardiac output, systemic inflammatory response, and endothelial dysfunction. Thromboembolic complications in HF patients most commonly lead to fatal consequences.

Methods: The protocol for this study was registered in PROSPERO with identification number CRD42024532861. We performed random-effects meta-analyses on extracted data of hazard ratios (HR) and odds ratios (OR) using Review Manager 5.4.

Results: Twenty-two studies with a total population of 14,773 patients were included. INR was associated with risk of mortality (per 0.1 increase, univariate HR = 1.17, 95% CI: 1.08–1.28, *p* = 0.0004; multivariate HR = 1.35, 95% CI: 0.80–2.29, *p* = 0.26). Increased D-dimer significantly increased risk of mortality (per 1 ng/mL increase; univariate HR = 1.87, 95% CI: 1.22–2.89, *p* = 0.004; multivariate HR = 1.90, 95% CI: 1.09–3.32, *p* = 0.02), and fibrinogen did not increase risk of mortality (per 1 mg/dL increase; HR = 0.93, 95% CI: 0.77–1.13, *p* = 0.45). Substantial heterogeneity was observed in D-dimer analyses. Cautious interpretation, noting 95% prediction intervals: 0.75–4.67 (univariate) and 0.58–6.19 (multivariate), should be implemented. Several analyses had insufficient data.

Conclusion: Low-certainty evidence weakly supports the potential of D-dimer as an independent prognostic marker in predicting HF mortality.

## 1. Introduction

Heart disease remains the number one cause of mortality in the United States^1^. Heart failure (HF) or congestive heart failure (CHF), defined by the American College of Cardiology (ACC) and American Heart Association (AHA), is a complex clinical syndrome with symptoms and signs that result from any structural or functional impairment of ventricular filling or ejection of blood^2^.

HF is one of the most common chronic diseases, with prevalence reaching 8.3% among older adults^3^, and poses a significant threat to global morbidity and mortality. Reports in 2017 estimated that HF was the cause of approximately 1.2 million hospitalizations^2^, with the average 1-year case fatality rate in HF being 33%^3^.

HF patients experience a wide range of physiological abnormalities, such as aberrant immune system activation, an elevated inflammatory response, altered endothelial function, excessive neuroendocrine activation, and abnormalities in the structure and function of the heart^4,5^. These physiological changes enhance the risk of forming blood clots, which follow Virchow’s triad: (1) stasis of blood flow due to poor contractility, (2) hypercoagulability caused by systemic inflammatory response, and (3) endothelial dysfunction^6^. Thromboembolic complications represent an annual prevalence of 1% and most commonly have fatal consequences^6^.

The objective of this meta-analysis was to systematically review and assess the possible association between abnormal coagulation factors and mortality risk in HF patients.

## 2. Methods

### 2.1. Protocol writing and registration

The protocol for this meta-analysis was registered prior to the start of the study in the International Prospective Register of Systematic Reviews (PROSPERO), with the identification number CRD42024532861. We reported the meta-analysis according to the guidelines of the Preferred Reporting Items for Systematic Reviews and Meta-Analyses (PRISMA) statement^7^.

### 2.2. Search

A literature search was conducted on 4 databases: PubMed/MEDLINE, ProQuest, Cochrane Library, and ScienceDirect on April 24, 2024. Detailed search strategies and the keywords searched in each database are attached (Supplementary Text S1). There were no additional articles added from other sources.

### 2.3. Study selection

Search results were compiled and deduplicated using Rayyan^8^. Two reviewers worked independently to screen abstracts using predetermined inclusion and exclusion criteria. The inclusion criteria for this meta-analysis were any observational studies (cross-sectional, case-control, or cohort) reporting on the association between abnormal coagulation parameters (prothrombin time (PT), international normalized ratio (INR), activated partial thromboplastin time (aPTT), D-dimer, fibrinogen) and HF mortality. We only included studies that reported odds ratios from logistic regression or hazard ratios from Cox regression models. Our prespecified primary outcome was mortality. We also included composite outcomes. Only articles published in English were included. Full text must be available. We did not apply restrictions on study designs or publication date. The exclusion criteria for this meta-analysis were review articles, editorials, letters, replies, systematic reviews, meta-analyses, studies on animals, and studies on children. We also excluded randomized controlled trials and other experimental studies given that the aim of these studies is to collect data related to the intervention rather than the association of coagulation factors with mortality. Conference abstracts were also excluded. No automation tool was used in the screening process.

An article would proceed to the full-text review stage if at least one reviewer considered it within the scope of the meta-analysis. Articles that passed the initial screening were sought for retrieval and reviewed by two reviewers working together.

### 2.4. Data extraction and quality assessment

Data were extracted on publication characteristics, baseline demographics, outcomes, and association of coagulation parameters with HF mortality in the forms of odds ratios (OR) or hazard ratios (HR). To better include all D-dimer studies, either using HR of each one ng/mL or specific cutoffs, we also collected D-dimer mean differences between survivors and non-survivors. Studies that reported median and interquartile range (IQR) values were converted to mean and standard deviation (SD) values using the following steps: (1) skewness of the data was detected using a method described by Shi et al.^9^; (2) mean values were estimated by using the method described by Luo et al.^10^, and SD values were estimated using the method described by Wan et al.^11^; (3) based on suggestions by Shi et al.^9^ on how to handle skewed data in analysis, we analyzed data with skewed distribution separately in a subgroup analysis. Two reviewers worked independently to extract data from the included papers and then compared and discussed any inconsistencies. A third reviewer was available if an extra opinion was needed to reach a consensus. Missing data were not sought further.

Risk of bias assessment was carried out by two reviewers working independently. Studies were assessed for quality using National Institutes of Health (NIH) Quality Assessment Tools^12^. Discrepancies between reviewers’ judgments were resolved by discussion with a third reviewer. A third reviewer attempted to resolve disagreements with an open discussion with the other two reviewers to agree on a score. If a score could not be agreed upon, the third reviewer would decide which of the two reviewers’ scores would be used. The results were included in the final report for the readers’ information.

### 2.5. Data synthesis and statistical analyses

All relevant study data and results were gathered and presented in tables. The minimum number of studies was two for each coagulation parameter. Study characteristics and findings were reported according to a standard format, and similarities and differences were compared across studies. If data were adequate, inverse variance random-effects meta-analyses were performed in Review Manager (Cochrane Collaboration). The inverse-variance method minimizes imprecision of the pooled effect estimate, based on standard errors^13^. The inverse-variance method could also be implemented directly using HRs and ORs from regression analyses. A random-effects model was selected considering that the included studies might have enough in common that it is plausible to synthesize the information, but are not truly identical^14^. The included studies were conducted by independent different researchers, with mixes of participants and methodological diversity, such that there may be different effect sizes in different studies^13,14^.

Forest plots were used during the meta-analysis to display the results. We interpreted a two-sided *p*-value of 0.05 as statistically significant. We explored statistical heterogeneity based on I^2^ values. An I^2^ value of 25% indicated low heterogeneity, an I^2^ value of 25–50% indicated moderate heterogeneity, and an I^2^ value of 50% indicated high heterogeneity. All results from the meta-analysis were published regardless of non-significant results.

### 2.6. Reporting bias and certainty assessment

We evaluated reporting bias using RoBANS 2: A Revised Risk of Bias Assessment Tool for Nonrandomized Studies of Interventions^15^. We used the Grading of Recommendations, Assessment, Development, and Evaluations (GRADE) to assess the certainty of the evidence^16^.

## 3. Results

### 3.1. Literature search

**Figure 1.**
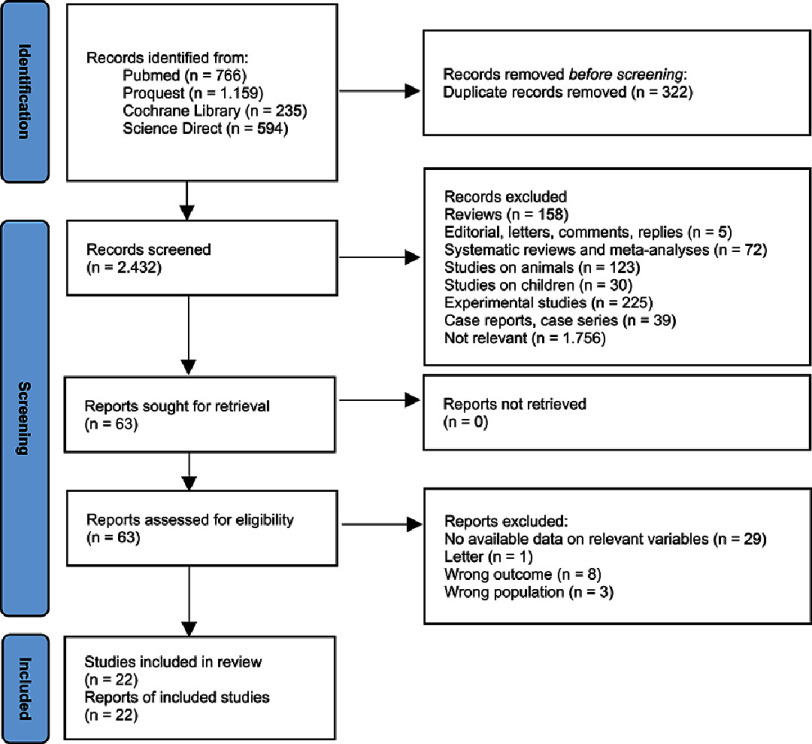
Article selection process flow diagram.

Literature search resulted in a total of 2,754 records across PubMed, ProQuest, Cochrane Library, and ScienceDirect. After deduplication, 2,432 distinct records remained and proceeded to our three-stage review process. The final 22 studies met the eligibility criteria and were included in the meta-analysis. A flow chart of the selection process and exclusion reasons is provided below (Figure 1).

### 3.2. Study characteristics

Twenty-two studies with a total population of 14,773 patients with publication years ranging from 2003-2024 were identified^17–38^. Most of the study designs were cohort studies, and the majority of the study locations were in China. The average age of the population was 61.62 years old, with 56% male and 44% female patients. The mean follow-up period of all studies was up to 654.68 days (we converted 1 year into 365 days and 1 month into 30 days, while studies that did not state a follow-up period were regarded as 0 days). Nine articles studied acute/decompensated HF patients, and 13 articles studied chronic/stable patients. Seventeen studies reported inpatient data, and 5 studies reported outpatient data. Table S1 summarized the study characteristics.

Two studies reported the association between PT and HF mortality, three reported the association between INR and HF mortality, while one study reported the association between aPTT and HF mortality. Fifteen studies reported the association between D-dimer and HF mortality, and five studies reported the association between fibrinogen and HF mortality. NIH Quality Assessment scores were presented along with the study characteristics in Table S1.

### 3.3. Prothrombin time and HF mortality

From the eligible studies, only the study by Tavares et al.^19^ and Lin et al.^34^ reported on the association between PT and HF mortality. Tavares et al. used a cutoff of PT > 14 s and found that patients with PT > 14 s were associated with higher 24-month mortality (HR = 1.88, 95% CI: 1.25–2.82, *p* = 0.002), meanwhile Lin et al. reported that each increase of 1 s in PT also increased 90-day mortality risk in chronic HF patients (OR = 1.005, 95% CI: 1.009–1.037, *p* = 0.001). We deemed that a meta-analysis was not appropriate due to the different measurement units reported in the studies.

### 3.4. International Normalized Ratio and HF mortality

**Figure 2.**
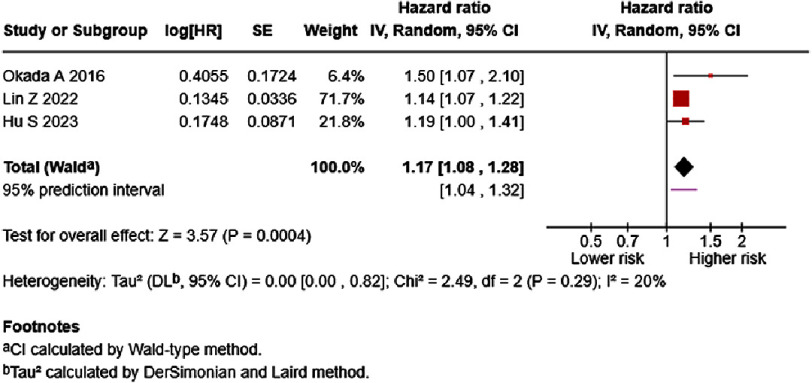
Effect of International Normalized Ratio and HF mortality (univariate). SE: standard error; IV: inverse-variance; CI: confidence interval; df: degree of freedom.

**Figure 3.**
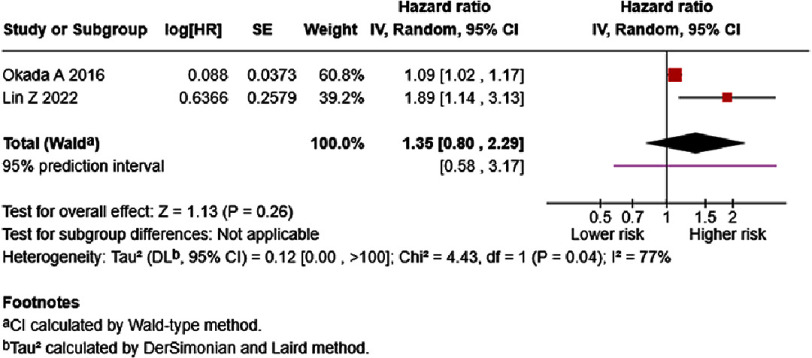
Effect of International Normalized Ratio and HF mortality (multivariate). SE: standard error; IV: inverse-variance; CI: confidence interval; df: degree of freedom.

Three studies reported the association between International Normalized Ratio (INR) and HF mortality^25,34,36^. The results of each study are presented in Table S2. In the univariate analysis, low heterogeneity (I2 = 20%) was observed, and higher INR significantly increased the risk for mortality in HF patients (per 0.1 increase, HR = 1.17, 95% CI: 1.08–1.28, *p* = 0.0004; Figure 2), with the 95% prediction interval showing a similar estimate (1.04–1.32). After adjustment for several confounding variables, the multivariate analysis of two studies showed that INR may increase the risk for mortality (per 0.1 increase HR = 1.35, 95% CI: 0.80–2.29, *p* = 0.26). High heterogeneity was observed (Figure 3). The 95% prediction interval showed a wider prediction range for future study results (0.58–3.17).

### 3.5. Activated partial thromboplastin time and HF mortality

Only one eligible study reported on the association between aPTT and HF mortality. Hu et al.^36^ found that higher aPTT significantly increased the risk for all-cause mortality (aPTT per 1 s increase, HR = 1.021, 95% CI: 1.003–1.039, *p* = 0.021).

### 3.6. D-dimer and HF mortality

**Figure 4.**
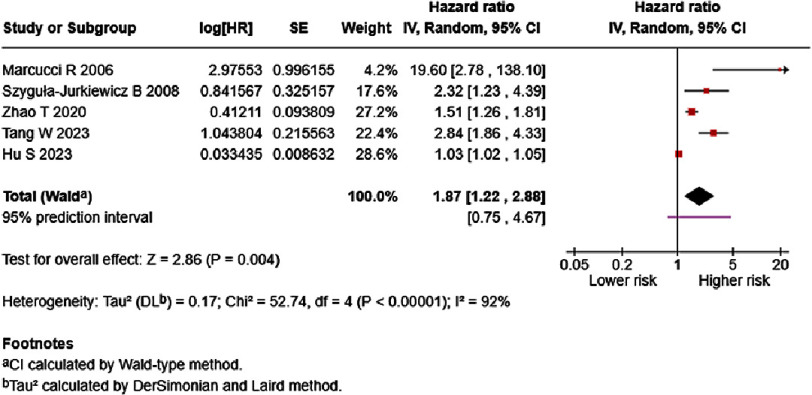
Effect of D-dimer and HF mortality (univariate). SE: standard error; IV: inverse-variance; CI: confidence interval; df: degree of freedom.

**Figure 5.**
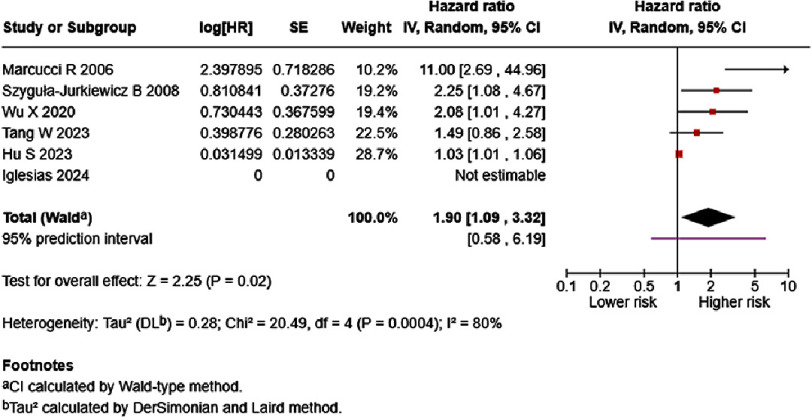
Effect of D-dimer and HF mortality (multivariate). SE: standard error; IV: inverse-variance; CI: confidence interval; df: degree of freedom.

Supplementary Table S3 present the reported results of fifteen studies which reported on the association between D-dimer and HF mortality ^18,20–23,26–32,35–37^. Eight studies, however, reported on variable D-dimer cut offs^18,22,23,26,27,29,31,32^. We performed a meta-analysis to calculate the pooled risk from studies which reported HR of each one ng/mL increase^20,21,28,30,35–37^. Evidence revealed that each one ng/mL increase of D-dimer significantly increased HF mortality (7 studies, 5,415 patients; univariate HR = 1.87, 95%CI: 1.22–2.89, *p* = 0.004; multivariate HR= 1.90, 95%CI: 1.09–3.32, *p* = 0.02; Figures 4 and 5). As high statistical heterogeneity was observed in both analyses, we are prompted to further analyze these results using prediction interval. Based on prediction interval calculations, 95% of future studies reporting association of D-dimer as a univariate in HF mortality will report HR that falls between 0.75–4.67, while 95% of future studies reporting association of D-dimer in multivariate analysis in HF mortality will report HR that falls between 0.58–6.19.

**Figure 6.**
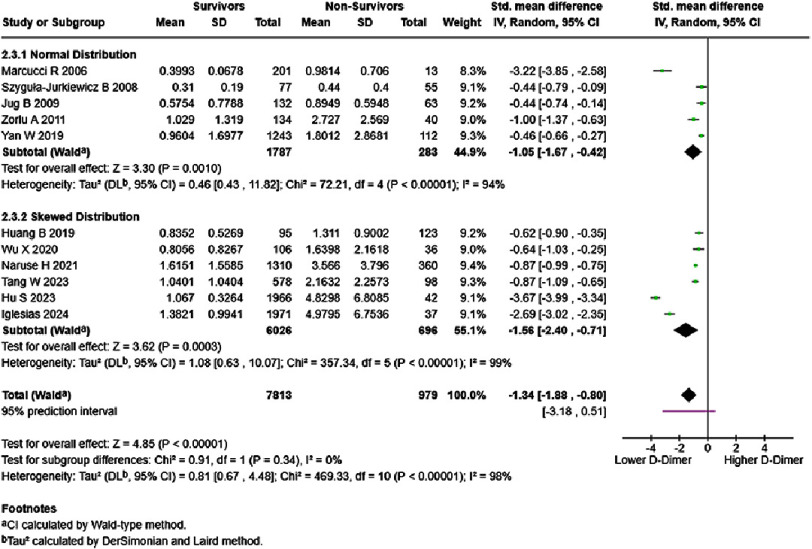
D-dimer levels in survivors vs non-survivors, in mg/dL. SE: standard error; IV: inverse-variance; CI: confidence interval; df: degree of freedom.

Eight studies reported on the association between D-dimer and HF mortality using cutoff values. Cutoff values used in studies ranged from 145–2600 ng/mL (median 840 ng/mL). All studies reported significantly higher mortality risk in patients with elevated D-dimer levels, with HR ranging from 1.25–3.23 (median HR 2.315). Results reported by each study were listed as an attachment (Table S3). Separate analysis of mean D-dimer difference levels was performed, as seen in Figure 6. Overall analysis showed statistically significant lower D-dimer levels in HF survivors (SMD = −1.34, 95% CI: −1.88 to −0.80, *p* < 0.00001), with both normal distribution subgroup (SMD = −1.05, 95% CI: −1.67 to −0.42, *p* < 0.00001) and skewed distribution subgroup (SMD = −1.56, 95% CI: −2.40 to −0.71, *p* = 0.0003) showing similar results, and no subgroup difference was detected.

**Figure 7.**
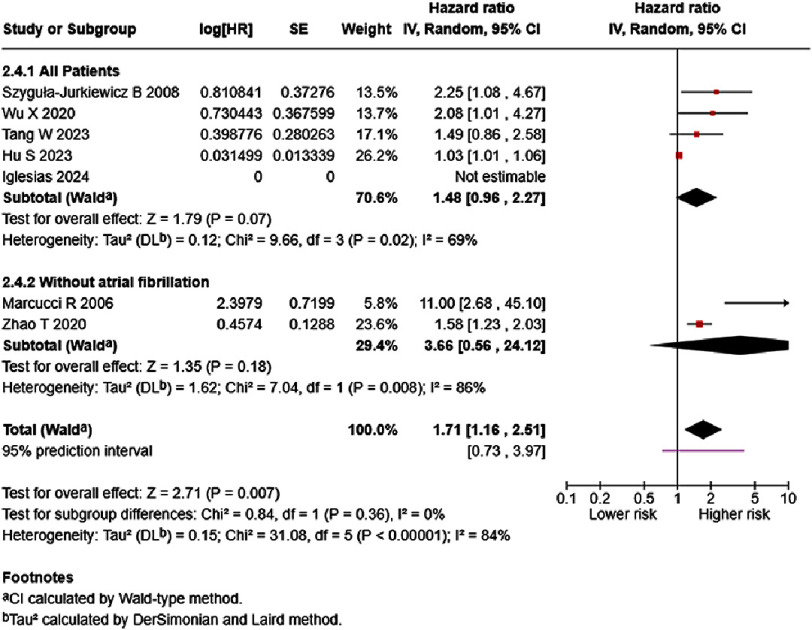
Subgroup analyses on the association between D-dimer and HF mortality on patients without atrial fibrillation. SE: standard error; IV: inverse-variance; CI: confidence interval; df: degree of freedom.

Subgroup analyses of HF patients without atrial fibrillation were undertaken (Figure 7); however, heterogeneity remained high and low subgroup differences were observed. The study by Iglesias et al. reported an HR close to 1 (HR = 1.00004, 95% CI: 1.0000–1.0001) and was not estimable in the software. The study by Zhao T et al.^30^ studied a subgroup of patients without atrial fibrillation, while the study by Marcucci et al.^20^ excluded patients with atrial fibrillation. The population without atrial fibrillation had higher but non-significant risk (HR = 3.66, 95% CI: 0.56–24.11, *p* = 0.18) than the general population (HR = 1.48, 95% CI: 0.96–2.27, *p* = 0.07). The meta-analysis indicated an overall significantly increased risk of mortality for each one ng/mL unit increase of D-dimer (HR = 1.71, 95% CI: 1.16–2.51, *p* = 0.007).

**Figure 8.**
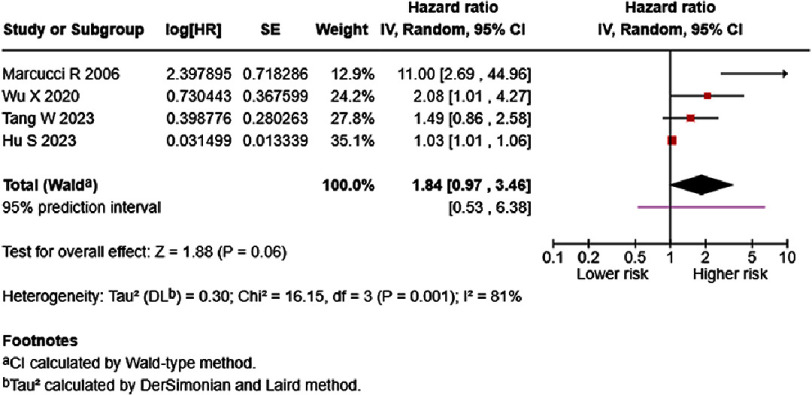
Effect of D-dimer and acute/decompensated HF mortality. SE: standard error; IV: inverse-variance; CI: confidence interval; df: degree of freedom.

**Figure 9.**
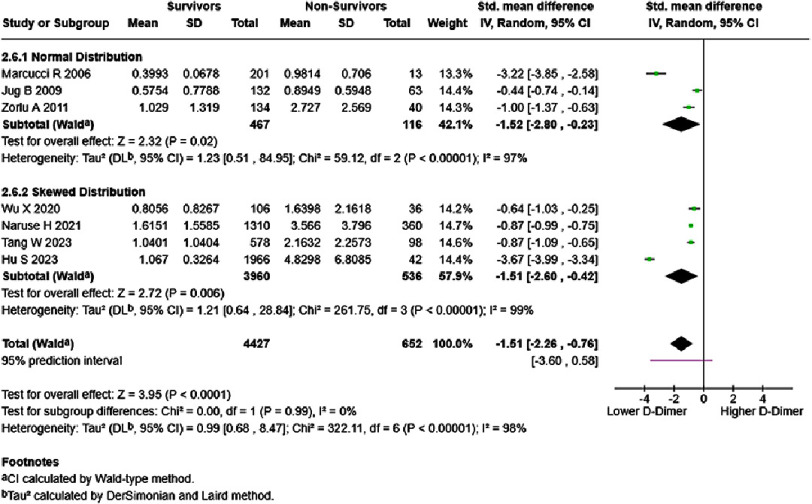
D-dimer levels in survivors vs non-survivors of acute/decompensated HF, in mg/dL. SE: standard error; IV: inverse-variance; CI: confidence interval; df: degree of freedom.

When grouped by onset, acute/decompensated HF patients possessed borderline significantly higher mortality risk (HR = 1.84, 95% CI: 0.97–3.46, *p* = 0.06) (Figure 8). Heterogeneity was classified as high, with a wide 95% prediction interval that crosses the line of null effect. Analyzing the difference in D-dimer levels of acute/decompensated HF patients showed significantly lower D-dimer levels in acute/decompensated HF survivors (SMD = −1.51, 95% CI: −2.26 to −0.76, *p* < 0.00001) (Figure 9). Additionally, analyses of both normal distribution (SMD = −1.52, 95% CI: −2.80 to −0.23, *p* < 0.00001) and skewed distribution groups (SMD = −1.51, 95% CI: −2.60 to −0.42, *p* < 0.00001) resulted in similar effect estimates, with high heterogeneity (97–99%).

**Figure 10.**
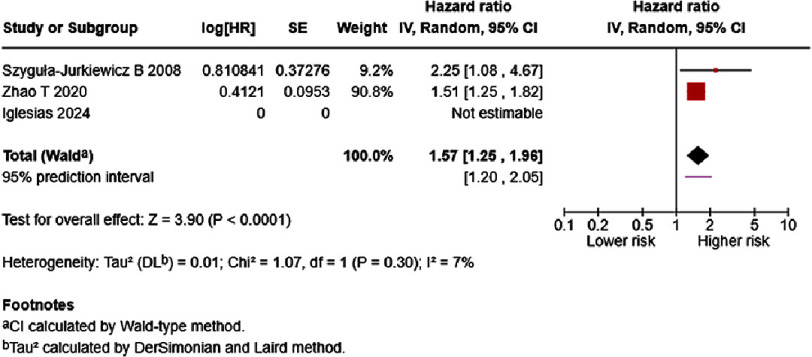
Effect of D-dimer and chronic/stable HF mortality. SE: standard error; IV: inverse-variance; CI: confidence interval; df: degree of freedom.

**Figure 11.**
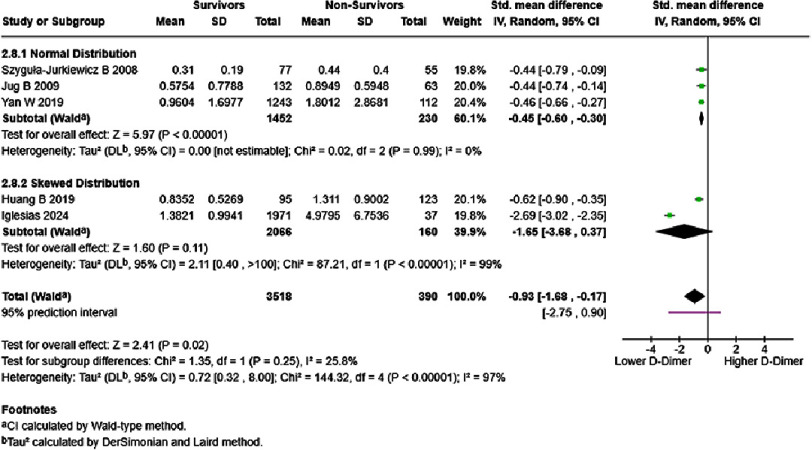
D-dimer levels in survivors vs non-survivors of chronic/stable HF, in mg/dL. SE: standard error; IV: inverse-variance; CI: confidence interval; df: degree of freedom.

In chronic/stable HF patients, D-dimer level of each one ng/mL significantly increased mortality risk (HR = 1.57, 95% CI [1.25–1.96], *p* < 0.0001), with a 95% prediction interval ranging from 1.20–2.05 (Figure 10). Low heterogeneity was noted; however, cautious interpretation was recommended due to the paucity of studies. Chronic/stable HF survivors possessed lower D-dimer levels than non-survivors (SMD = −0.93, 95% CI: −1.68 to −0.17, *p* = 0.02). Differences in effect estimates, significance, and heterogeneity between normal distribution (SMD = −0.45, 95% CI: −0.60 to −0.30, *p* < 0.00001) and skewed distribution groups (SMD = −1.65, 95% CI: −3.68 to 0.37, *p* = 0.11) could be seen (Figure 11).

**Figure 12.**
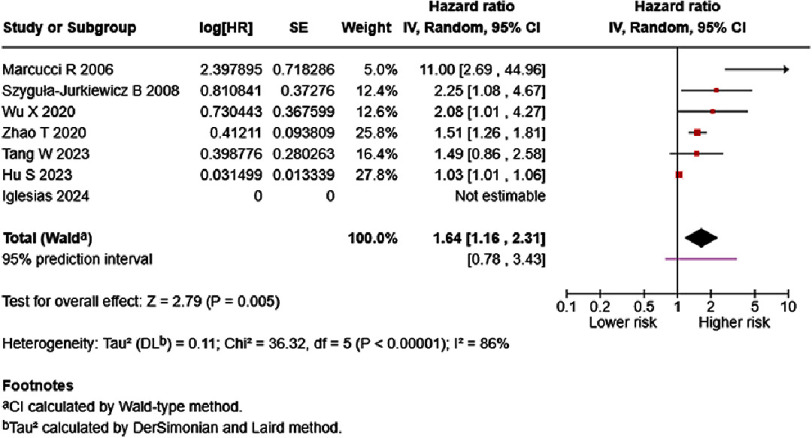
Effect of D-dimer and inpatient HF mortality. SE: standard error; IV: inverse-variance; CI: confidence interval; df: degree of freedom.

**Figure 13.**
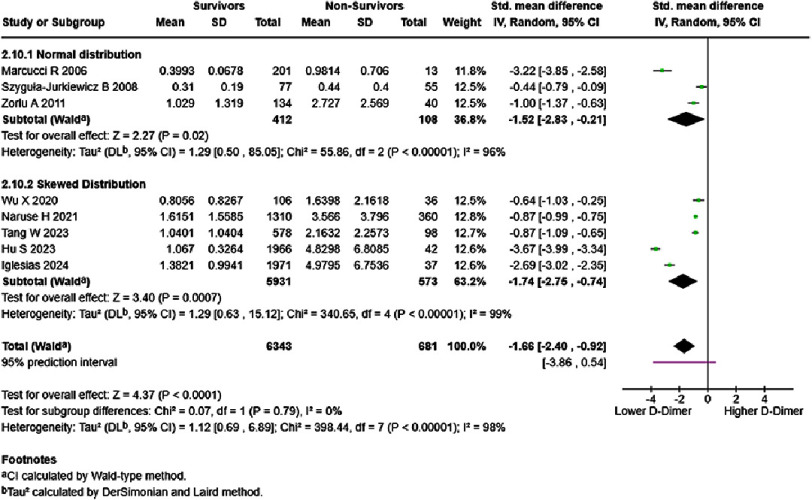
D-dimer levels in survivors vs non-survivors of inpatient HF, in mg/dL. SE: standard error; IV: inverse-variance; CI: confidence interval; df: degree of freedom.

**Figure 14.**
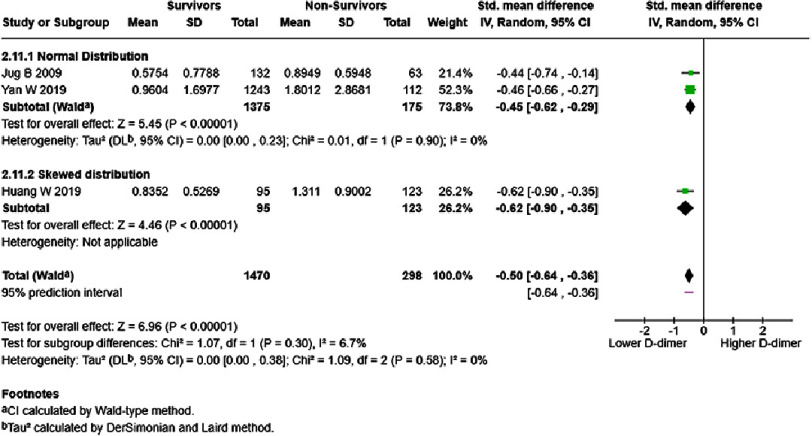
D-dimer levels in survivors vs non-survivors of outpatient HF, in mg/dL. SE: standard error; IV: inverse-variance; CI: confidence interval; df: degree of freedom.

In analyses based on inpatient/outpatient groups, D-dimer level of each one ng/mL significantly increased mortality risk in HF inpatients (HR = 1.64, 95% CI: 1.16–2.31, *p* < 0.005). High heterogeneity was observed, with a 95% prediction interval ranging from 0.78–3.43 (Figure 12). HF inpatient survivors possessed lower D-dimer levels than non-survivors (SMD = −1.66, 95% CI: −2.40 to −0.92, *p* < 0.0001) (Figure 13). No subgroup difference was detected. HF outpatient survivors possessed lower D-dimer levels than non-survivors (SMD = −0.50, 95% CI: −0.64 to −0.36, *p* < 0.00001). No subgroup difference was observed; however, low heterogeneity was noted (Figure 14).

### 3.7. Fibrinogen and HF mortality

**Figure 15.**
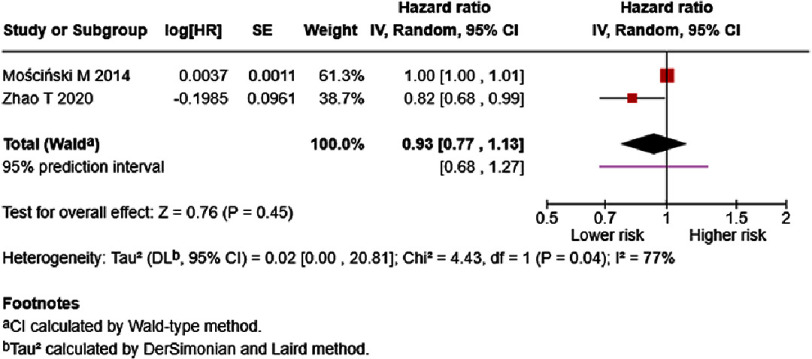
Effect of fibrinogen and HF mortality (Univariate). SE: standard error; IV: inverse-variance; CI: confidence interval; df: degree of freedom.

Table S4 presents results from five studies on fibrinogen and HF mortality. Three studies reported data in variable formats, while two studies reported data for each 1 mg/dL increase in fibrinogen. The study by Meng et al.^33^ reported that high fibrinogen group (≥284 mg/dL) patients were at increased risk of mortality (HR = 3.11, 95% CI: 1.81–5.36, *p* < 0.0001), even after adjusting for confounding variables. From a meta-analysis of two studies, results displayed that a 1 mg/dL increase in fibrinogen may reduce the risk of mortality (HR = 0.93, 95% CI: 0.77–1.13, *p* = 0.45, Figure 15). High heterogeneity was observed.

### 3.8. Reporting bias and evidence grading

Based on RoBANS 2 reporting bias evaluation, we found the studies to be of low risk of reporting bias. Evidence grading using the GRADE approach yielded various levels among all analyses. The authors considered the grading of the evidence on D-dimer effect on HF mortality to be low certainty. Serum D-dimer may be useful as an individual prognostic marker to predict mortality in HF patients, but our confidence in the effect is limited. We took into consideration the number of studies and the consistent direction of effect between studies. However, persistent heterogeneity and the diverse population and methods in all studies might introduce biased results in the meta-analyses. Evidence on PT, INR, aPTT, and fibrinogen was considered to be very low certainty. This was largely due to the paucity of studies and insufficient data to draw a definite conclusion. It might be reasonable to measure these coagulation parameter levels in HF patients; however, usefulness is unknown. The rationale for each GRADE domain assessment is provided in a table adapted from Prasad and the Cochrane Handbook of Systematic Reviews of Interventions^13,39^ (Supplementary Text S2).

## 4. Discussion

Our meta-analysis found low-certainty evidence highlighting the potential of D-dimer as an independent prognostic marker in predicting HF mortality, and very low-certainty evidence suggesting that increased INR may increase mortality risk in patients with HF. It is important to note that the INR analysis had consequential concerns related to the paucity of eligible studies. The other three analyses on PT, aPTT, and fibrinogen yielded inconclusive results.

Meta-analysis of each one ng/mL D-dimer increase demonstrated significantly increased mortality risk (HR = 1.90, 95% CI: 1.09–3.32, *p* = 0.02), with high heterogeneity and a 95% prediction interval crossing the line of null effect, ranging from 0.58–6.19. We explored the robustness of the result by excluding results from each study. The results remained significant even when excluding the study by Marcucci et al., due to the much larger effect estimates that deviated from the rest of the studies (HR = 1.45, 95% CI: 1.06–1.98, *p* = 0.02). Supplementary analyses to assess the robustness of findings are available (Supplementary Figures).

Subgroup analyses based on presence of atrial fibrillation showed lower effect magnitudes that were not statistically significant and provided little explanation for heterogeneity. Separate analyses of both onset and hospital admission shed some light on possible causes of heterogeneity. Analysis of D-dimer and chronic/stable HF mortality risk showed I^2^ = 7%, with SMD of D-dimer in survivors vs. non-survivors in chronic/stable HF with normal distribution resulting in I^2^ = 0%. Outpatient HF analysis using SMDs resulted in I^2^ = 0%. These results could be interpreted to mean that D-dimer association with acute/decompensated HF and HF inpatients might observe a wider range of effects, while studies on chronic/stable HF and HF outpatients observed results that fairly overlap with each other. Nevertheless, explorations of heterogeneity at best only lead to the generation of hypotheses^13^. Secondly, too few studies are included in the chronic/stable and outpatient analyses, leading to questionable value.

High statistical heterogeneity was a cause for concern in our meta-analysis. We underwent several steps to explore this issue^13^. First, we rechecked all entered data. Second, high heterogeneity might mean that a meta-analysis is not recommended if the studies are too diverse. However, I^2^ is a means to measure statistical heterogeneity and not clinical or methodological diversity^13^. From the clinical and methodological aspects, we deemed the included studies still addressed our review objective, and it is generally reasonable to conduct a meta-analysis as a way to estimate effect size in a wide population^40^. Third, heterogeneity may be a consequence of inappropriate choice of effect measure; however, we ensured that our data used the same effect measures. Fourth, sensitivity analyses were conducted, but heterogeneity remained. Fifth, subgroup analyses were undertaken; however, they did not explain heterogeneity completely and could only lead to hypotheses, as stated above. Sixth, a random-effects model was chosen instead of a fixed-effect model. Fixed-effect meta-analyses ignore heterogeneity; however, considerable heterogeneity suggests that there is a variety of intervention effects. A random-effects meta-analysis incorporates heterogeneity among studies and is intended for heterogeneity that cannot be explained^13^.

A random-effects meta-analysis model assumes the results follow some distribution, calculated using a 95% prediction interval. A common criticism is the difficulty in establishing the validity of any distributional assumption^13^. High heterogeneity means effects are expected to differ from one patient to another, with some experiencing increased mortality risk, some experiencing minimal increase in mortality risk, while others did not experience any increase at all^40^. For example, although the present meta-analysis resulted in each one ng/mL increase of D-dimer significantly increasing mortality risk (HR = 1.90, 95% CI: 1.09–3.32, *p* = 0.02), the probable distribution of effect ranges from 0.58–6.19. These estimates should be taken into consideration when interpreting the results.

In studies that used cutoff D-dimer values, all studies consistently reported that increased mortality was observed in the higher D-dimer group. With the purpose of detecting potential bias from excluding these studies, we conducted additional sensitivity analyses using mean and SD values. Comparison of the forest plots showed no visibly significant differences between studies using cutoffs and studies using per unit values, with overall analysis resulting in statistically significant lower D-dimer levels in HF survivors (SMD = −1.34, 95% CI: −1.88 to −0.80, *p* < 0.00001). There are two things to note in this analysis. First, some studies reported median and IQR values with skewed data distribution; thus, the estimated mean and SD values might be imprecise^10^. To mitigate the effect of this bias, we followed the suggestion of Shi et al. to analyze normal data distribution and skewed data distribution in subgroups^9^. Testing for subgroup differences resulted in no significant subgroup differences. Second, not every included D-dimer study provided comparison of D-dimer levels in survivors vs. non-survivors. Potential biases due to missing data cannot be ruled out.

D-dimer as a continuous variable might be useful in comparing mortality risk between patients with different D-dimer levels. Meta-analysis of each unit measure was also possible despite unreported individual participant data. However, it is also reasonable to consider D-dimer value as a cutoff variable. The presence of normal limits of D-dimer suggests that a certain level of D-dimer is physiological^41^, and using D-dimer as a continuous variable to calculate mortality risk under such conditions might not be appropriate. As all studies did not report individual participant data, no reliable method could combine HRs of various cutoffs of D-dimer as a prognostic marker for HF mortality. Exclusion of studies using D-dimer cutoff values might lead to overestimation of mortality risk even when D-dimer levels are well within normal limits. Clinicians should take both measures into account when estimating a patient’s mortality risk.

An interesting finding to note was the consistent direction of effect across multiple studies, which, despite the low certainty of evidence, suggests a potential role for D-dimer as a prognostic biomarker. Clinically, elevated D-dimer levels may help identify HF patients at potentially higher risk of mortality, warranting closer monitoring or more individualized management. Based on our findings, several included studies applied D-dimer cutoff values ranging from 145 to 2600 ng/mL, with a median threshold of approximately 840 ng/mL increasing mortality risk in HF patients with HR = 2.315 (95% CI: 1.570–3.414), *p* < 0.001. This median value was not universally standardized and should be approached carefully, ideally in conjunction with other clinical indicators due to the limited data. Future studies should evaluate this value prospectively to determine its sensitivity and specificity in HF populations.

A large retrospective cohort combined HFpEF and HFrEF patients as its population in the investigation of coagulation factor abnormalities and mortality^31^. Elevated D-dimer, HF risk score, and NT-proBNP were each independently associated with 12-month mortality across both phenotypes (all *p* < 0.05). These findings suggest that both HFpEF and HFrEF may manifest similar systemic procoagulant activity^31^. Merging both subtypes enhances the generalizability of prognostic models and reflects the clinical reality in acute settings where left ventricular ejection fraction (LVEF) may not be immediately known.

This meta-analysis included studies with markedly different follow-up periods, ranging from days to years. Combining studies with such variability in follow-up duration may impact the interpretation of prognostic markers, as short-term mortality often reflects acute hemodynamic disturbances or complications, while long-term outcomes may be influenced by chronic disease progression and comorbidities^42^. For example, based on our analysis, D-dimer as a prognostic biomarker has lower inconsistency in chronic/stable HF than in acute/decompensated HF. The study by Alehagen et al.^18^ supports this distinction, showing that elevated baseline D-dimer was an independent predictor of cardiovascular death over a 4-year period in HF outpatients. The prognostic strength was most apparent in the follow-up, suggesting D-dimer’s utility may be time-dependent. The heterogeneity in follow-up periods may also dilute pooled effect sizes and reduce the evidence certainty, underscoring the need to interpret results in the context of follow-up duration.

D-dimer as a cost-effective biomarker in detecting mortality risk in HF patients primarily has strengths such as its relatively low cost compared to other prognostic tools such as natriuretic peptides^30^. While a formal cost-effectiveness analysis was beyond the scope of our meta-analysis, a previous study reported that D-dimer monitoring holds promise^30^. When applied selectively, especially in patients at higher risk of thromboembolic complications or poor outcomes, D-dimer testing could support early risk identification, guide closer clinical monitoring, and help prioritize resources for those likely to benefit from intensified care^43–45^.

Several studies are in line with the results of our meta-analysis. D-dimer has been associated with worse outcomes in numerous major cardiovascular diseases^46^. A previous study found that plasma D-dimer significantly predicted thromboembolic events and mortality independently among coronary artery disease (CAD) patients^47^, while two other studies found D-dimer was useful to identify mortality risk in severe hypertension patients^48^ and dilated cardiomyopathy^49^. Another relevant study found increased D-dimer levels were associated with increased incidence of ischemic stroke in acute HF patients^46^. HF patients are prone to develop pathophysiological coagulation mechanisms due to myocardial myopathy, increased vascular stiffness, myocardial ischemia, and low cardiac output^50^. D-dimer has been found to correlate with levels of BNP, which reflected worse severity in HF patients, translating to poorer survival outcomes^5^.

Meta-analysis of INR value was mostly obtained from very low certainty evidence that points to possible reduction of mortality in HF patients. Univariate analysis of INR showed significant results; however, multivariate analysis displayed an insignificantly increased risk for each 0.1 increase. The effects of INR on mortality risk remain to be clarified, with very weak recommendation as a prognostic marker in HF patients. Analysis of fibrinogen yielded conflicting results between studies, with meta-analysis demonstrating that fibrinogen was a protective factor against mortality, but several studies reported otherwise. More studies should be conducted on other coagulation factors to provide substantial conclusive evidence.

Future research recommendations include analyses of each D-dimer unit association with mortality risk after a specific threshold, for example each one ng/mL increase after a threshold of 500 ng/mL. These studies may be able to provide better understanding of existing studies, possibly being an intermediary between studies using unit values and cutoff values. Additionally, it is recommended that future studies analyze different HF populations in subgroups as a way to minimize heterogeneity.

### 4.1. Strengths and limitations

Although the role of coagulation factors in HF has been discussed in previous studies, this meta-analysis is the first to systematically investigate and analyze effect estimates of coagulation factors, primarily D-dimer. We made efforts to ensure each process in the meta-analysis resulted in as low a risk of bias as possible, for example, using multiple reviewers and blinding where applicable. The main limitation is unexplained heterogeneity that seemed to be observable in most analyses, despite our several efforts. Two possible explanations were that there was an unexplored cause of heterogeneity or interstudy heterogeneity, e.g., resulting from different researchers and patients. Some have argued that heterogeneity is inevitable^14,51^, and about a quarter of meta-analyses have I^2^ values over 50%^51^. The presence of substantial heterogeneity should be considered, including the clinical implications of the said heterogeneity across studies. Interpretation of heterogeneity could differ according to whether the estimates had the same direction of effect^51^. Presenting 95% prediction intervals for the readers’ information minimizes overconfidence in the evidence.

We considered performing meta-regression; however, this was not feasible due to an insufficient number of studies per covariate. Sources have suggested that meta-regression should generally not be considered when there are fewer than ten studies in a meta-analysis^13^. While our total D-dimer studies reached 15, these studies reported different outcome measures, resulting in all meta-analyses not having more than 10 studies, therefore limiting the possibility of meta-regression. Another limitation to note was the lack of sufficient data to reliably measure the effect magnitude of PT, INR, aPTT, and fibrinogen on HF mortality (Table 1).

**Table 1:** Summary of key findings.

Variable	Results	Certainty of evidence	Concerns
PT	Inconclusive	Very low certainty	Severely inadequate data to draw a conclusion
INR	Increased INR may be associated with mortality risk of HF patients	Very low certainty	Paucity of studies
aPTT	Inconclusive	Very low certainty	Severely inadequate data to draw a conclusion
D-dimer	Increased D-dimer level may be associated with increased mortality risk in HF patients	Low certainty	Substantial heterogeneity
Fibrinogen	Mixed results, inconclusive	Very low certainty	Paucity of studies

## 5. Conclusion

D-dimer may be useful as an independent prognostic factor in predicting HF mortality, with low-certainty evidence and weak recommendation due to high heterogeneity between studies. Its clinical utility warrants cautious interpretation.

## Acknowledgements

We thank our family and friends for their support during the production of this article.

### Author statement

Conceptualization: Ceria Halim.

Data curation: Ceria Halim.

Formal analysis: Ceria Halim, Billy P. Teruna, Indah R. Harahap, Eric T. Fernando, and L. B. C. Nugroho.

Funding acquisition: Ceria Halim, Billy P. Teruna, Indah R. Harahap, Eric T. Fernando, and L. B. C. Nugroho.

Investigation: Billy P. Teruna and Indah R. Harahap.

Methodology: Ceria Halim.

Project administration: L. B. C. Nugroho.

Resources: Billy P. Teruna.

Software: Ceria Halim.

Supervision: Ceria Halim.

Validation: Eric T. Fernando and L. B. C. Nugroho.

Visualization: Ceria Halim.

Writing - original draft: Ceria Halim, Billy P. Teruna, and Indah R. Harahap.

Writing - review & editing: Ceria Halim and Eric T. Fernando.

### Funding

This research received no external funding.

## Competing interests

The authors declare no conflict of interest.

### Patient consent for publication

Not required.

### Ethics approval

Ethics approval was not needed as the study used already published data.

### Data availability statement

Data is contained within the article or supplementary material.

### Supplemental material

Available via journal website.
